# Female Sex Is a Risk Factor Associated with Long-Term Post-COVID Related-Symptoms but Not with COVID-19 Symptoms: The LONG-COVID-EXP-CM Multicenter Study

**DOI:** 10.3390/jcm11020413

**Published:** 2022-01-14

**Authors:** César Fernández-de-las-Peñas, José D. Martín-Guerrero, Óscar J. Pellicer-Valero, Esperanza Navarro-Pardo, Víctor Gómez-Mayordomo, María L. Cuadrado, José A. Arias-Navalón, Margarita Cigarán-Méndez, Valentín Hernández-Barrera, Lars Arendt-Nielsen

**Affiliations:** 1 Department of Physical Therapy, Occupational Therapy, Physical Medicine and Rehabilitation, Universidad Rey Juan Carlos (URJC), 28922 Alcorcón, Spain; 2 CNAP, Center for Sensory-Motor Interaction (SMI), Department of Health Science and Technology, Faculty of Medicine, Aalborg University, 9220 Aalborg, Denmark; lan@hst.aau.dk; 3 Intelligent Data Analysis Laboratory, Department of Electronic Engineering, ETSE (Engineering School), Universitat de València (UV), 46100 Valencia, Spain; Jose.d.martin@uv.es (J.D.M.-G.); oscar.pellicer@uv.es (Ó.J.P.-V.); 4 Department of Developmental and Educational Psychology, Universitat de València (UV), 46010 València, Spain; esperanza.navarro@uv.es; 5 Department of Neurology, Hospital Universitario Clínico San Carlos, 28040 Madrid, Spain; vicmayordomo@gmail.com (V.G.-M.); mlcuadrado@med.ucm.es (M.L.C.); 6 Department of Medicine, School of Medicine, Universidad Complutense de Madrid, 28040 Madrid, Spain; 7 School of Health Sciences, Universidad Alfonso X El Sabio, 28691 Madrid, Spain; josari@uax.es; 8 Department of Psychology, Universidad Rey Juan Carlos (URJC), 28933 Madrid, Spain; margarita.cigaran@urjc.es; 9 Department of Public Health, Universidad Rey Juan Carlos (URJC), 28933 Madrid, Spain; valentin.hernandez@urjc.es; 10 Department of Medical Gastroenterology, Aalborg University Hospital, 9100 Aalborg, Denmark

**Keywords:** COVID-19, sex, female, symptoms, fatigue, post-COVID, risk factors

## Abstract

This multicenter cohort study investigated the differences between coronavirus disease 2019 (COVID-19) related symptoms and post-COVID symptoms between male and female COVID-19 survivors. Clinical and hospitalization data were collected from hospital medical records in a sample of individuals recovered from COVID-19 at five public hospitals in Spain. A predefined list of post-COVID symptoms was systematically assessed, but patients were free to report any symptom. Anxiety/depressive levels and sleep quality were also assessed. Adjusted multivariate logistic regressions were used to identify the association of sex with post-COVID related-symptoms. A total of 1969 individuals (age: 61, SD: 16 years, 46.4% women) were assessed 8.4 months after discharge. No overall significant sex differences in COVID-19 onset symptoms at hospital admission were found. Post-COVID symptoms were present in up to 60% of hospitalized COVID-19 survivors eight months after the infection. The number of post-COVID symptoms was 2.25 for females and 1.5 for males. After adjusting by all variables, female sex was associated with ≥3 post-COVID symptoms (adj OR 2.54, 95%CI 1.671–3.865, *p* < 0.001), the presence of post-COVID fatigue (adj OR 1.514, 95%CI 1.040–2.205), dyspnea (rest: adj OR 1.428, 95%CI 1.081–1.886, exertion: adj OR 1.409, 95%CI 1.109–1.791), pain (adj OR 1.349, 95%CI 1.059–1.720), hair loss (adj OR 4.529, 95%CI 2.784–7.368), ocular problems (adj OR 1.981, 95%CI 1.185–3.312), depressive levels (adj OR 1.606, 95%CI 1.002–2.572) and worse sleep quality (adj OR 1.634, 95%CI 1.097–2.434). Female sex was a risk factor for the development of some long-term post-COVID symptoms including mood disorders. Healthcare systems should consider sex differences in the management of long haulers.

## 1. Introduction

The world has experienced a dramatic situation due to the rapid spread of the severe acute respiratory syndrome coronavirus 2 (SARS-CoV-2) causing the coronavirus disease 2019 (COVID-19). It has been reported that the COVID-19 outbreak has led to increased gender inequality [1]. Therefore, recognizing the extent to which COVID-19 affect women and men differently is an important step to better understand the pathophysiology and nature of COVID-19 sequalae with the aim to promote personalized healthcare interventions [2].

There is evidence showing that men and women exhibit the same probability of being infected by SARS-CoV-2; however, males are 2.4 at a higher risk of death than females at the acute infection [3,4,5,6]. Older age, male sex, and the presence of comorbidities are independent risk factors associated with higher in-hospital mortality of COVID-19 patients [7].

There is an increasing interest in the relevance of symptoms presented at the acute and post-COVID phase. Current evidence supports that symptoms associated with SARS-CoV-2 infection are heterogeneous and affect different systems [8], but there is a lack of studies specifically investigating potential sex differences in COVID-19-associated symptoms. Similarly, although different meta-analyses provide prevalence rates of post-COVID symptoms ranging from 35% to 60% depending on the symptom and the follow-up period [9,10,11,12], sex differences are not clearly defined. Two meta-analyses have tried to identify risk factors potentially associated with the development of post-COVID symptoms; however, the number of studies was limited, and the results related to sex differences were conflicting [13,14]. For instance, some studies found that females are more prone to develop post-COVID-19 symptoms than males [15,16,17,18,19], whereas others did not find such association [20,21,22,23,24,25]. It should be noted that most studies included samples with <500 participants, were conducted in one single center, included follow-up periods shorter than 12 weeks and did not specifically focus on sex differences [15,16,17,18,19,20,21,22,23,24,25]. Three recent multicenter studies have been published [26,27,28]. Sigfrid et al. included 327 hospitalized COVID-19 survivors from five hospitals and reported that females younger than 50 years were two times more likely to report fatigue and seven times more likely to report dyspnea than men of the same age seven months after hospital discharge [26]. Munblit et al. included 2649 COVID-19 survivors from four different hospitals and found that female sex was associated with persistent post-COVID symptoms, particularly dermatological changes, seven months after hospitalization [27]. Zhang et al. included 2433 hospitalized COVID-19 survivors from two centers and reported that female sex was associated with a higher risk of post-COVID fatigue [28].

We present here a multicenter study (LONG-COVID-EXP-CM) aimed to specifically investigate sex differences on COVID-associated symptoms and long-term post-COVID symptoms in a sample of previously hospitalized COVID-19 survivors in a large Spanish population. We hypothesized that females would exhibit similar symptoms during the acute phase of the SARS-CoV-2 infection but greater post-COVID symptoms than males.

## 2. Methods

### 2.1. Participants

The LONG-COVID-EXP-CM is a multicenter cohort study including individuals hospitalized due to SARS-CoV-2 infection (ICD-10 code) during the first wave of the pandemic (from 10 March to 31 May 2020) in five hospitals of Madrid (Spain) associated with the Spanish National Health Service. All diagnoses were conducted with real-time reverse transcription-polymerase chain reaction (PCR-RT) assay of nasopharyngeal/oral swab samples and presence of positive radiological findings at hospital admission. All COVID-19 survivors discharged from the participating hospitals were included in an anonymous electronic database and a total random selection of 400 individuals from each hospital was performed by using Excel^®^ version 2021 (Microsoft 365©). The study was approved by the Local Ethics Committees of all involved institutions (HUFA 20/126, URJC0907202015920, HCSC20/495E, HUIL/092-20, HUF/EC1517, HSO25112020). Participants provided their informed consent before collecting any data. It should be noted that the same sample of participants included in the LONG-COVID-EXP-CM study has been used in three letters to the editor [29,30,31], but current data are completely new and not previously published.

### 2.2. COVID-19 and Post-COVID-19 Related-Symptoms Data Collection

The current analysis used a cross-sectional design by collecting data of the patients at hospital admission and at a single follow-up period.

Participants who agreed to participate were scheduled for a telephone interview by trained healthcare researchers following the procedures used in population-based survey studies. A specific questionnaire for the current study was developed by a multidisciplinary research team. Participants were systematically asked for the presence of any symptom from a predefined list of post-COVID symptoms, e.g., fatigue, dyspnea (at rest or exertion), anosmia, ageusia, hair loss, throat pain, diarrhea, palpitations, cough, cognitive blunting (brain fog), skin rashes, memory loss, visual disorders, voice problems, gastrointestinal disturbances, pain symptoms, or concentration loss. Participants were free to report any other symptom not included in the list and that they suffered from. It was emphasized that symptoms should have appeared after hospital discharge (post-COVID-19 related symptoms). More than one symptom could be reported by the same participant and the number of symptoms was calculated.

Clinical (age, sex, height, weight, pre-existing medical comorbidities) and hospitalization (symptoms at hospital admission, days at hospital, intensive care unit (ICU) admission) data were collected from hospital medical records.

### 2.3. Psychological Data Collection

The presence of anxiety/depressive symptoms and quality of sleep were assessed with the Hospital Anxiety and Depression Scale (HADS) and Pittsburgh Sleep Quality Index (PSQI), respectively, since both questionnaires can be properly executed by telephone interview [32]. The HADS includes one subscale assessing anxiety symptoms (HADS-A, 7-items, 0–21 points) and another one assessing depressive symptoms (HADS-D, 7-items, 0–21 point) [33]. Although a cut-off score of ≥8 points has shown good sensitivity and specificity to determine the presence of anxiety or depressive symptoms [34], we used the cut-off scores recommended for the Spanish population indicative of anxiety (HADS-A ≥12 points) and depressive (HADS-D ≥10 points) symptoms [35]. The HADS has shown good validity and reliability, and it has been previously used in patients with COVID-19 [36].

The PSQI (0–21 points) evaluates sleep quality by including 19 self-rated questions assessing aspects such as usual bedtime, usual wake time, number of hours slept, and number of minutes to fall asleep [37]; scores ≥8.0 points suggest poor sleep quality [37]. The PSQI has shown good internal consistency and test–retest reliability [38].

### 2.4. Statistical Analysis

For the initial binary statistical analysis, sex (male, female) was the independent variable, whereas clinical, COVID-19 symptoms at hospital admission, post-COVID symptoms, and psychological data were the dependent variables. Missing values were imputed using median imputation. For binary variables (data presented as percentages) sex differences were analyzed with the Chi-squared test, while for remaining of the variables (presented as means with standard deviations, SD), *t* tests were employed. Holm–Bonferroni was used for correcting the *p* values for this analysis. Accordingly, multivariate logistic regressions were conducted to identify the independent association of sex with variables significantly different between males and females in the binary analysis: (1) headache as an onset symptom at hospital admission adjusted by the following variables: age, height, weight, pre-existing medical comorbidities; (2) post-COVID symptoms adjusted by all variables collected at hospital admission (age, height, weight, pre-existing medical comorbidities, COVID-19 onset symptoms at hospital admission, intensive care unit (ICU) admission, days at hospital) by using Python’s library statsmodels 0.11.1 and Scipy 1.6.2 (Python Software Foundation, Wilmington, DE, USA). Gender was always an independent variable as well as all variables collected at hospital admission and an intercept term. Accordingly, adjusted odds ratio (OR) and their confidence intervals (95%CI) were calculated for headache as onset symptom and post-COVID variables. For continuous variables (number of post-COVID symptoms, HADS-A, HADS-D and PSQI), the following classification was used: ≥3 number of post-COVID symptoms; HADS-A ≥ 12 points; HADS-D ≥ 10 points; PSQI ≥ 8 points to define anxiety, depression, and poor sleep quality, respectively. A priori, a level of significance of 0.05 was considered.

## 3. Results

### 3.1. Participants

From a total of 7150 patients hospitalized at the participating hospital during the first wave of the pandemic (10 March to 31 May 2020), 2000 participants were randomly selected and invited to participate. Six refused participation, eleven were not contacted after three attempts, and fourteen had deceased after hospital discharge. A total of 1969 participants (mean age: 61, SD: 16 years, 46.4% women) were finally included.

### 3.2. COVID-19 Symptoms and Comorbidities at Hospital Admission and Sex

The most common symptoms at hospital admission due to SARS-CoV-2 infection included fever (74.6%), dyspnea (31.5%), myalgia (30.65%), and cough (27.9%). The only COVID-19 onset symptom at hospital admission significantly different between females and males was headache: a greater proportion of females (20.7%) experienced headache as COVID-19 onset symptom (*p* = 0.003) when compared with males (13.5%, Table 1).

Eight hundred and thirty-six (*n* = 836, 42.5%) did not report medical comorbidities, 717 (36.4%) had one comorbidity, 283 (14.4%) had two comorbidities, and the remaining 133 (6.7%) had three comorbidities. No sex differences in the number of pre-existing medical comorbidities (*p* = 0.989) were observed. A greater proportion of females reported pre-existing asthma, musculoskeletal pain, and rheumatological conditions (all, *p* < 0.001).

### 3.3. Post-COVID-19 Symptoms, Anxiety, Depression, Sleep Quality and Sex

Participants were assessed a mean of 8.4 months (SD, 1.5) after hospital discharge. Thirty-seven percent (*n* = 737, 37.4%) were completely free of any post-COVID symptom, whereas 283 (14.4%) had ≥3 post-COVID symptoms. The most frequent post-COVID symptoms were fatigue (61.3%) and dyspnea at exertion (53.5%). The number of post-COVID symptoms was significantly higher (*p* < 0.001) in females (mean: 2.25, SD: 1.4) than in males (mean: 1.5, SD: 1.3).

Similarly, a greater proportion of females reported the presence of some post-COVID symptoms including fatigue, dyspnea, pain symptoms, ocular problems, and hair loss when compared with males (Table 2). In general, females showed higher total scores (all, *p* < 0.001) in the HADS-A, HADS-D and PSQI questionnaires than males (Table 2).

**Table 1:** Clinical and hospitalization data according to sex.

	Total (*n* = 1969)	Female (*n* = 915)	Male (*n* = 1054)	*p* Value
Age (years)	61.1 ± 16.3	60.5 ± 17.1	61.6 ± 15.5	0.145
Weight (kg) *	74.8 ± 15.3	68.7 ± 14.8	80.1 ± 13.8	<0.001
Height (cm) *	165.0 ± 16.5	158.1 ± 16.3	170.7 ± 14.7	<0.001
Number of pre-existing co-morbidities	0.85 ± 0.9	0.85 ± 0.8	0.85 ± 0.9	0.758
Obesity (pre-existing)	88 (4.5%)	35 (3.8%)	53 (5%)	0.208
Hypertension (pre-existing)	514 (26.1%)	222 (24.2%)	292 (27.7%)	0.136
Diabetes (pre-existing)	236 (12.0%)	101 (11.0%)	135 (12.8%)	0.258
Asthma (pre-existing) *	126 (6.4%)	84 (9.2%)	42 (4%)	<0.001
COPD (pre-existing)	77 (3.9%)	28 (3.05%)	49 (4.6%)	0.07
Musculoskeletal Pain (pre-existing) *	806 (40.9%)	443 (48.4%)	363 (34.4%)	<0.001
Cardiac diseases (pre-existing)	234 (11.9%)	92 (10.05)	142 (13.5%)	0.762
Rheumatological diseases (pre-existing) *	31 (1.6%)	30 (3.3%)	1 (0.001%)	<0.001
Other diseases (pre-existing)	332 (16.9%)	179 (19.5%)	153 (14.5%)	0.222
Number of symptoms at hospital admission	2.2 ± 0.8	2.2 ± 0.7	2.15 ± 0.8	0.662
Fever (COVID-19 onset)	1469 (74.6%)	642 (70.1%)	827 (78.4%)	0.857
Dyspnea (COVID-19 onset)	620 (31.5%)	277 (30.3%)	343 (32.5%)	0.371
Myalgias (COVID-19 onset)	604 (30.65%)	310 (33.9%)	294 (27.9%)	0.536
Cough (COVID-19 onset)	549 (27.9%)	248 (27.1%)	301 (28.55%)	0.542
Headache (COVID-19 onset) *	332 (16.9%)	190 (20.7%)	142 (13.5%)	0.003
Diarrhea (COVID-19 onset)	210 (10.65%)	113 (12.3%)	97 (9.2%)	0.857
Anosmia (COVID-19 onset)	167 (8.5%)	84 (9.2%)	83 (7.9%)	0.321
Ageusia (COVID-19 onset)	145 (7.35%)	68 (7.4%)	77 (7.3%)	0.918
Throat pain (COVID-19 onset)	102 (5.2%)	48 (5.25%)	54 (5.1%)	0.905
Vomiting (COVID-19 onset)	55 (2.8%)	35 (3.8%)	20 (1.9%)	0.353
Dizziness (COVID-19 onset)	66 (3.35%)	32 (3.5%)	34 (3.2%)	0.742
Days at hospital	11.3 ± 11.4	10.6 ± 10.7	11.8 ± 11.9	0.651
ICU admission	130 (6.6%)	57 (6.2%)	73 (7%)	0.548

COPD: chronic obstructive pulmonary disease; ICU: intensive care unit; * significant differences between males and females (*p* < 0.05).

**Table 2:** Post-COVID symptoms and psychological symptoms according to sex.

	Total (*n* = 1969)	Female (*n* = 915)	Male (*n* = 1054)	*p*-Value
Time after hospital discharge	8.4 ± 1.5	8.35 ± 1.5	8.45 ± 1.5	0.845
Number of post-COVID symptoms *	1.9 ± 1.4	2.25 ± 1.4	1.55 ± 1.3	<0.001
≥3 post-COVID symptoms *	647 (32.85%)	402 (43.9%)	245 (23.25%)	<0.001
Fatigue *	1206 (61.3%)	623 (68.1%)	583 (55.3%)	0.01
Dyspnea at rest *	459 (23.3%)	257 (28.1%)	202 (19.15%)	0.002
Dyspnea at exertion *	1054 (53.5%)	548 (59.9%)	506 (48.0%)	0.01
Pain Symptoms (including headache) *	887 (45.1%)	461 (50.4%)	426 (40.4%)	0.035
Memory Loss	341 (17.3%)	169 (18.5%)	172 (16.3%)	0.252
Cognitive Blurring-Frain Fog	189 (9.6%)	99 (10.8%)	90 (8.5%)	0.103
Concentration Loss	140 (7.1%)	75 (8.2%)	65 (6.2%)	0.101
Hair Loss *	470 (23.9%)	341 (37.3%)	129 (12.2%)	<0.001
Palpitations-Tachycardia	140 (7.1%)	79 (8.6%)	61 (5.8%)	0.544
Skin Rashes	236 (12%)	128 (14%)	108 (10.25%)	0.536
Gastrointestinal Problems	133 (6.75%)	66 (7.2%)	67 (6.35%)	0.465
Diarrhea	49 (2.5%)	25 (2.7%)	24 (2.3%)	0.523
Voice Problems	35 (1.8%)	18 (2%)	17 (1.6%)	0.556
Ageusia	53 (2.7%)	29 (3.2%)	24 (2.3%)	0.229
Anosmia	80 (4.05%)	46 (5.0%)	34 (3.2%)	0.143
Ocular Problems *	116 (5.9%)	73 (8%)	43 (4.1%)	0.01
Throat Pain	50 (2.5%)	24 (2.6%)	26 (2.5%)	0.828
HADS-A (0–21) *	4.9 ± 5.3	5.5 ± 5.2	4.3 ± 5.3	<0.001
Anxiety (HADS-A ≥12 points)	308 (15.6%)	147 (16.1%)	161 (15.3%)	0.658
HADS-D (0–21) *	4.7 ± 4.8	5.2 ± 5.1	4.2 ± 4.5	<0.001
Depression (HADS-D ≥10 points) *	373 (18.9%)	217 (23.7%)	156 (14.8%)	<0.001
Sleep Quality (0–21) *	6.5 ± 4.0	7.3 ± 4.2	5.8 ± 3.7	<0.001
Poor Sleep Quality (PSQI ≥8 points) *	674 (34.2%)	383 (41.9%)	291 (27.6%)	<0.001

HADS: Hospital Anxiety and Depression Scale (A: anxiety; D: depression); PSQI: Pittsburgh Sleep Quality Index; * significant differences between males and females (*p* < 0.05).

### 3.4. Multivariate Analysis

The first multivariate analysis revealed that female sex was not independently and significantly associated with headache as an onset symptom at hospital admission (adj OR 1.323, 95%CI 0.972–1.801, *p* = 0.075) after adjusting by variables collected at hospitalization.

The second multivariate analysis, after adjusting by all variables collected at hospital admission revealed that female sex was significantly associated with ≥3 post-COVID symptoms (adj OR 2.54, 95%CI 1.671–3.865, *p* < 0.001), and the presence of post-COVID fatigue (adj OR 1.514, 95%CI 1.040–2.205, *p* = 0.017), dyspnea (rest: adj OR 1.428, 95%CI 1.081–1.886, *p* = 0.012; exertion: adj OR 1.409, 95%CI 1.109–1.791, *p* = 0.005), pain (adj OR 1.349, 95%CI 1.059–1.720, *p* = 0.016), hair loss (adj OR 4.529, 95%CI 2.784–7.368, *p* < 0.001) or ocular problems (adj OR 1.981, 95%CI 1.185–3.312, *p* = 0.009). Additionally, the multivariate analysis also found that female sex was significantly associated with depressive levels (adj OR 1.606, 95%CI 1.002–2.572, *p* = 0.045) and worse sleep quality (adj OR 1.634, 95%CI 1.097–2.434, *p* = 0.004), but not with anxiety levels (adj OR 1.145, 95%CI 0.733–1.788, *p* = 0.998).

Supplementary Tables S1 and S2 show the adjusted OR of all variables collected at hospital admission (which were adjusted for in the multivariate analysis) related to those post-COVID symptoms, including mood disorders, which were significantly associated with female sex. As can be observed in the Supplementary Tables, age, height, weight, pre-existing medical comorbidities, COVID-19 onset symptoms at hospital admission, intensive care unit (ICU) admission or days at hospital were not significantly overall associated with those long-term post-COVID symptoms or mood disorders significantly associated with female sex. The only significant additional association to female sex was the presence of pre-exiting pain symptoms for some of post-COVID symptoms.

## 4. Discussion

This multi-center cohort study specifically investigating sex differences in the presence of COVID-19 onset-symptoms and long-term post-COVID symptoms in previously hospitalized COVID-19 survivors found that COVID-19 symptoms at hospital admission were similar between males and females; however, females were more prone to develop post-COVID symptomatology eight months after hospital discharge than males.

The topic of female sex as a potential risk factor for developing post-COVID symptomatology has been previously suggested in the literature [13,14]; however, results are contradictory since a small number of studies supports this association [15,16,17,18,19], whereas others did not [20,21,22,23,24,25]. Recent studies including larger cohort samples from only a single center also found that female sex was a risk factor associated with post-COVID syndrome [39,40]. Additionally, in agreement with our results, three multicenter studies also reported that female sex is a potential risk factor for some post-COVID symptoms, e.g., fatigue, dyspnea or dermatological symptoms [26,27,28]. 

Nevertheless, previous studies did not specifically investigate sex differences, and they did not compare differences in COVID-19-associated onset symptoms. Our study is the largest specifically exploring the role of sex on the development of post-COVID symptoms. Accordingly, considering sex differences in diagnosis, prevention and treatment of diseases are fundamental steps toward precision medicine [41]. Based on our results, females are more vulnerable to develop post-COVID symptoms; therefore, sex and gender differences should be considered when managing individuals who had recovered from COVID-19 and develop post-COVID sequelae [42].

Other risk factors suggested in the literature to be associated with post-COVID symptoms include age, greater number of COVID-19 symptoms at the acute phase, longer hospital stance and the number of medical comorbidities [13,14]. It has been previously suggested that older age is a risk factor associated with post-COVID symptoms [43,44,45]. We did not find such association after adjusting for hospital admission variables since age was not significantly associated with post-COVID symptoms in our analysis. Similarly, we did not find overall sex differences in COVID-19-associated symptoms at hospital admission (only that the presence of headache as an onset symptom was more prevalent in females than in male) nor within hospital stay. Similarly, although the presence of some medical comorbidities, such as hypertension, diabetes, or cardiovascular diseases, have been associated with a higher risk of severe illness or death at the acute phase of the infection, again, we did not find an association of pre-existing medical co-morbidities with post-COVID symptoms. Preliminary evidence suggests that previous medical comorbidities, e.g., asthma, are not associated with post-COVID symptoms [46,47]. It seems that female sex would be a risk factor associated with the presence of long-term post-COVID symptoms but not with COVID-19 onset symptoms.

Different underlying mechanisms explaining why females can develop post-COVID symptoms to a greater extent than males are currently discussed in the literature. First, biological differences on the expression of angiotensin-converting enzyme-2 (ACE2) and transmembrane protease serine 2 (TMPRSS2) receptors between males and females, and immunological differences, e.g., lower production of pro-inflammatory interleukin-6 (IL-6) after viral infection in females, could explain the higher development of post-COVID symptoms [48,49]. Second, sanitary-related behaviors, e.g., more frequent hand washing or less exposure in females, have been also considered for explaining sex differences [50]; however, these factors would unlikely explain the fact that females are at higher risk of developing post-COVID symptoms. As the prevalence of pain syndromes is higher in females (the presence of pre-existing pain was also associated with some of the post-COVID symptoms associated with sex), it is also possible that other factors such as higher psychological stress could play a role in the development of post-COVID symptoms. We found that females also exhibited higher depressive levels and poor sleep quality than males eight months after hospital discharge. Evidence supports the presence of anxiety, depression and sleep disorders in the general population due to COVID-19 pandemic [51]. It is possible that COVID-19 outbreak surrounding factors such as isolation, stress, inactivity, could also promote the development of more post-COVID symptoms in females.

Although this is a large multicenter study investigating sex differences in post-COVID symptoms, some limitations are recognized. First, we only included hospitalized COVID-19 survivors; therefore, these data should not be extrapolated to non-hospitalized patients. Second, because we used a cross-sectional design, we are not able to determine the evolution of post-COVID symptoms from hospital discharge. Third, we only included Caucasian participants; therefore, extrapolation of current findings to other ethnicities should be not performed. Fourth, several psychological (e.g., post-traumatic stress), social (e.g., isolation, stigmatization), or familiar (e.g., infection or death of a familiar) stressors, which could influence the presence of some post-COVID symptoms were not evaluated. Finally, we mostly collected self-reported patient outcomes, but not objective measures, e.g., blood oxygen saturation, inflammatory biomarkers, or chest X-ray, which could help to further identify sex differences in future studies.

## 5. Conclusions

This multicenter study revealed that post-COVID symptoms are present in up to 60% of hospitalized COVID-19 survivors eight months after infection. No significant sex differences in COVID-19 onset symptoms at hospital admission were seen. Females were at a higher risk for developing long-term post-COVID symptoms including anxiety, depression, or poor sleep quality than males. Healthcare systems should consider sex differences in the management of long haulers.

## Data Availability

All data derived from this analysis is presented in the text.

## Supplementary Materials

The following supporting information can be downloaded at: https://www.mdpi.com/article/10.3390/jcm11020413/s1, Table S1: Adjusted odd ratio, 95% confidence interval, of baseline variables used in the multivariate analysis in post-COVID symptoms. Table S2: Adjusted odd ratio, 95% confidence interval, of baseline variables used in the multivariate analysis for depressive levels and poor sleep quality.
