# Supplementary material for: Female Sex Is a Risk Factor Associated with Long-Term Post-COVID Related-Symptoms but Not with COVID-19 Symptoms: The LONG-COVID-EXP-CM Multicenter Study
Source: J Clin Med. 2022 Jan 14;11(2):413. doi: 10.3390/jcm11020413 (PMC8778106; doi:10.3390/jcm11020413)
Supplement: Supplementary file 1 [file jcm-11-00413-s001.zip › jcm-1534974-supplementary.pdf]

Journal of Clinical Medicine

MDPI

Supplementary Table S1: Adjusted odd ratio, 95% confidence interval, of baseline variables used in the multivariate analysis in post-COVID symptoms.

	Fatigue	Dyspnea at rest	Dyspnea exertion	Pain Symptoms	Hair Loss	Ocular Problems	≥3 post-COVID symptoms
Age (years)	1.003, 0.993-1.013	1.002, 0.992-1.012	1.003, 0.993-1.013	0.995, 0.988-1.002	0.978, 0.956-1.002	0.991, 0.971-1.011	1.000, 0.992-1.009
Weight (kg)	1.002, 0.990-1.014	0.998, 0.984-1.011	1.002, 0.990-1.013	1.010, 0.997-1.024	1.003, 0.990-1.017	1.010, 0.990-1.031	1.001, 0.990-1.012
Height (cm)	0.993, 0.973-1.013	0.981, 0.957-1.006	0.992, 0.973-1.012	0.992, 0.978-1.005	1.004, 0.982-1.026	0.997, 0.963-1.032	1.000, 0.982-1.019
Obesity (pre-existing)	2.038, 0.548-7.581	3.321, 0.565-19.52	3.059, 0.783-11.94	2.478, 0.631-9.726	0.459, 0.096-2.200	1.743, 0.337-9.201	1.996, 0.500-7.967
Hypertension (pre-existing)	2.084, 0.686-6.334	2.052, 0.435-9.681	2.295, 0.732-7.196	1.996, 0.626-6.365	1.060, 0.371-3.029	1.156, 0.208-6.425	1.616, 0.492-5.312
Diabetes (pre-existing)	1.926, 0.609-6.086	2.114, 0.423-10.55	1.653, 0.518-5.270	1.404, 0.455-4.336	0.566, 0.142-2.247	0.467, 0.078-2.800	0.848, 0.276-2.600
Asthma (pre-existing)	3.321, 0.949-11.627	5.004, 0.954-26.25	5.899, 0.924-27.59	2.703, 0.761-9.607	0.784, 0.212-2.903	0.478, 0.070-3.258	2.353, 0.635-8.719
COPD (pre-existing)	3.317, 0.857-12.835	4.171, 0.771-22.58	4.533, 0.950-17.87	2.372, 0.635-8.854	0.382, 0.072-2.024	0.923, 0.170-4.999	1.463, 0.396-5.405
Musculoskeletal Pain (pre)	1.549, 1.119-2.145	1.144, 0.813-1.611	1.496, 1.094-2.047	1.553, 1.271-1.898	1.376, 0.951-1.992	1.411, 0.770-2.586	1.492, 1.067-2.085
Cardiac diseases (pre-existing)	2.255, 0.700-7.264	3.365, 0.658-17.22	3.189, 0.949-10.71	2.463, 0.725-8.365	1.020, 0.331-3.144	0.530, 0.075-3.770	1.714, 0.478-6.140
Rheumatological diseases (pre)	4.846, 0.642-36.581	0.986, 0.239-4.068	0.974, 0.240-4.099	2.669, 0.510-13.96	0.679, 0.121-3.819	0.484, 0.066-3.524	0.845, 0.203-3.511
Other diseases (pre-existing)	2.045, 0.650-6.434	2.572, 0.528-12.53	2.464, 0.727-8.024	2.052, 0.625-6.738	1.113, 0.363-3.412	0.381, 0.020-7.117	2.509, 0.712-8.833
Number symptoms at admission	4.095, 0.605-27.713	1.361, 0.420-3.138	1.374, 0.337-5.602	2.339, 0.528-10.36	7.560, 1.379-41.45	1.017, 0.259-3.990	3.199, 0.684-14.96
Fever (COVID-19 onset)	0.364, 0.053-2.505	5.631, 0.569-55.76	1.050, 0.279-3.952	0.486, 0.109-2.175	0.163, 0.029-0.922	0.867, 0.506-1.486	0.457, 0.098-2.144
Dyspnea (COVID-19 onset)	0.507, 0.075-3.411	4.183, 0.430-40.67	1.544, 0.358-6.660	0.680, 0.163-2.844	0.216, 0.039-1.194	1.073, 0.535-2.150	0.629, 0.141-2.809
Myalgias (COVID-19 onset)	0.341, 0.049-2.373	2.423, 0.263-22.29	0.944, 0.267-3.335	0.780, 0.191-3.188	0.176, 0.032-1.001	1.618, 0.691-3.788	0.456, 0.097-2.132
Cough (COVID-19 onset)	0.278, 0.040-1.939	3.189, 0.345-29.48	0.852, 0.218-3.328	0.529, 0.119-2.353	0.158, 0.28-0.877	1.310, 0.556-3.034	0.625, 0.090-2.004
Headache (COVID-19 onset)	0.384, 0.054-2.722	2.858, 0.297-27.49	1.101, 0.277-4.385	0.934, 0.261-3.346	0.273, 0.050-1.506	1.724, 0.709-4.191	0.600, 0.127-2.841
Diarrhea (COVID-19 onset)	0.284, 0.039-2.084	3.087, 0.316-30.13	0.843, 0.201-3.533	0.681, 0.153-3.039	0.257, 0.044-1.486	1.661, 0.616-4.482	0.641, 0.135-3.051
Anosmia (COVID-19 onset)	0.296, 0.041-2.143	1.543, 0.164-14.51	0.904, 0.242-3.378	0.426, 0.090-2.018	0.174, 0.029-1.063	1.389, 0.455-4.236	0.284, 0.055-1.468
Ageusia (COVID-19 onset)	0.423, 0.056-3.188	2.648, 0.255-27.53	0.862, 0.217-3.424	0.585, 0.123-2.777	0.193, 0.032-1.176	1.387, 0.423-4.547	0.847, 0.200-3.585
Throat pain (COVID-19 onset)	0.372, 0.048-2.882	2.584, 0.252-26.44	0.822, 0.183-3.687	0.864, 0.199-3.746	0.304, 0.050-1.846	1.673, 0.478-5.857	0.742, 0.158-3.477
Vomiting (COVID-19 onset)	0.404, 0.049-3.359	3.659, 0.319-42.03	0.920, 0.282-2.996	0.556, 0.109-2.849	0.176, 0.026-1.210	2.106, 0.458-9.672	0.517, 0.095-2.811
Dizziness (COVID-19 onset)	0.276, 0.034-2.244	1.995, 0.197-20.17	0.755, 0.164-3.468	0.679, 0.140-3.280	0.218, 0.033-1.428	1.197, 0.292-4.912	0.542, 0.102-2.884
Days at hospital	1.004, 0.991-1.017	1.018, 0.999-1.030	1.014, 0.999-1.028	1.012, 0.998-1.026	1.022, 0.999-1.039	1.011, 0.987-1.037	1.016, 0.999-1.030
ICU admission	0.904, 0.500-1.636	1.433, 0.736-2.787	0.853, 0.466-1.562	1.482, 0.791-2.776	1.027, 0.579-1.821	0.601, 0.158-2.276	1.169, 0.625-2.187

Journal of
Clinical Medicine

Supplementary Table S2: Adjusted odd ratio, 95% confidence interval, of baseline variables used in the multivariate analysis for depressive levels and poor sleep quality.

	**Depression (HADS-D≥10 points)**	**Poor Sleep Quality (PSQI ≥ 8 points)**
Age (years)	1.004, 0.991-1.016	1.003, 0.993-1.014
Weight (kg)	1.007, 0.992-1.022	1.006, 0.993-1.019
Height (cm)	0.979, 0.952-1.006	0.985, 0.963-1.007
Obesity (pre-existing)	0.551, 0.109-2.780	1.107, 0.357-3.434
Hypertension (pre-existing)	1.131, 0.328-3.900	0.915, 0.353-2.373
Diabetes (pre-existing)	1.184, 0.356-3.939	0.726, 0.247-2.133
Asthma (pre-existing)	1.227, 0.342-4.404	1.416, 0.436-4.598
COPD (pre-existing)	1.039, 0.324-3.331	0.707, 0.211-2.366
Musculoskeletal Pain (pre)	0.984, 0.765-1.265	1.519, 1.098-2.102
Cardiac diseases (pre-existing)	0.872, 0.238-3.193	1.189, 0.397-3.564
Rheumatological diseases (pre)	0.739, 0.129-4.238	0.628, 0.131-3.017
Other diseases (pre-existing)	1.478, 0.381-5.734	1.195, 0.415-3.438
Number symptoms at admission	6.041, 1.254-29.098	0.966, 0.292-3.197
Fever (COVID-19 onset)	0.269, 0.054-1.327	0.995, 0.344-2.883
Dyspnea (COVID-19 onset)	0.279, 0.057-1.361	1.465, 0.354-6.060
Myalgias (COVID-19 onset)	0.215, 0.044-1.050	1.332, 0.300-5.919
Cough (COVID-19 onset)	0.154, 0.031-0.773	1.115, 0.306-4.065
Headache (COVID-19 onset)	0.185, 0.036-1.005	1.397, 0.300-6.504
Diarrhea (COVID-19 onset)	0.207, 0.039-1.097	1.374, 0.309-6.099
Anosmia (COVID-19 onset)	0.226, 0.042-1.210	1.155, 0.270-4.942
Ageusia (COVID-19 onset)	0.248, 0.046-1.339	1.628, 0.355-7.914
Throat pain (COVID-19 onset)	0.139, 0.023-0.831	1.871, 0.367-9.537
Vomiting (COVID-19 onset)	0.160, 0.023-1.131	1.388, 0.275-7.009
Dizziness (COVID-19 onset)	0.147, 0.023-1.001	1.679, 0.322-8.768
Days at hospital	1.017, 0.999-1.030	1.022, 1.007-1.038
ICU admission	1.820, 0.917-3.612	1.407, 0.751-2.633
